# The Trade‐Off of Delivery Timing Between Reduced Perinatal Complications Versus Adverse Long‐Term Outcomes

**DOI:** 10.1111/ppe.70025

**Published:** 2025-04-20

**Authors:** Dieter Wolke, Xuan Zhao, Siobhan Quenby

**Affiliations:** ^1^ Department of Psychology, Lifespan Health and Wellbeing Group University of Warwick Coventry UK; ^2^ Warwick Medical School University of Warwick Coventry UK

Over the last decades, there has been an increase in studies on the impact of the timing of delivery at term on the long‐term outcome of babies. Randomised controlled trials (RCTs) have mainly focused on short‐term outcomes ranging from perinatal mortality to required respiratory support [1] and indicate positive effects on short‐term outcomes. In contrast, longer‐term observational studies reported that early‐term birth may negatively affect long‐term developmental outcomes [2].

This issue of *Paediatric and Perinatal Epidemiolog*y includes a carefully planned and executed study by Nguyen and colleagues [3], which utilised linked registry data from Sweden and British Columbia, Canada, adding to the picture that delivery at early term (37 or 38 weeks gestation) is associated with a risk of increased rates of adverse neurodevelopmental outcomes. They report that those born at 40 weeks gestation are at the lowest risk of attention deficit hyperactivity disorder (ADHD), while rates are up to 10% increased in those born early term. The study has many strengths. It is based on two large population cohorts in different countries with different obstetric practices and careful controls for a range of medical and social confounders, including parental neurodevelopmental disorders. They were also able to consider the mode of delivery. The effects of early‐term birth on ADHD appeared marginally higher in those who experienced induction of labour and planned caesarean delivery. However, this must be carefully interpreted due to the less precise effect estimates (wide 95% confidence intervals).

## 1 Re‐Balancing Short‐Term Benefits With Potential Long‐Term Trade‐Offs

Obstetricians recommend early induction of labour because of current or previous pregnancy complications and to reduce serious perinatal adverse outcomes. NHS England and the Royal College of Obstetricians and Gynaecologists [4] have amended obstetric practice to encourage induction of labour from 39 weeks in pregnancies identified as ‘at risk’ of stillbirth. Most research evidence that focuses on immediate perinatal outcomes supports this advice. A Cochrane review published in 2020 [1] that included 34 RCTs (reporting on over 21,000 women and infants) found that early induction after 37 weeks significantly reduced perinatal mortality, caesarean rates, 5‐min Apgar score less than 7, and NICU admissions compared to expectant management. Another Cochrane review [5] showed that early induction of labour was associated with a lower risk of shoulder dystocia and bone fractures in suspected foetal macrosomia, that is, large babies. The NHS policy of induction of labour is considered to have contributed to a reduction of the stillbirth rate in England and Wales from 5.6 to 4.0 (2001–2023) per 1000 live births [6].

**FIGURE 1:**
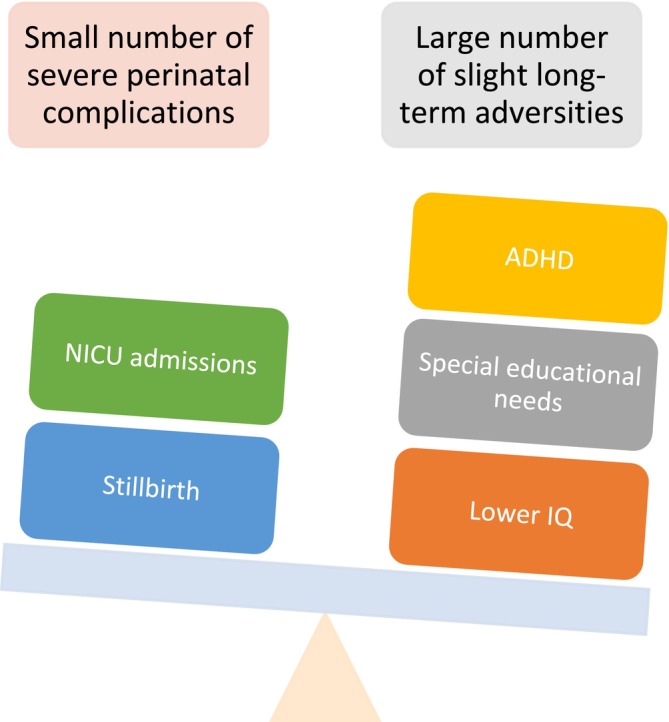
The trade‐off of delivery timing. ADHD, attention deficit hyperactivity disorder; IQ, intelligence quotient; NICU, neonatal intensive care unit.

However, clinical decisions regarding labour timing on perinatal outcomes may be short‐sighted if they do not consider long‐term consequences. Existing clinical trials have only short‐term follow‐up periods and ignore potential consequences for child neurodevelopment, cognitive, or academic outcomes. Evidence from a meta‐analysis [2] that included 62 observational studies suggests that children born at 37 weeks had lower cognitive scores (−1.95 IQ points), a higher risk of cognitive impairment, and poorer academic performance than children born at 40 weeks. Compared with children born at full‐term, those born at early‐term had a higher risk of ADHD and other cognitive impairments [2]. The carefully controlled study by Nguyen et al. confirms these results for ADHD. Based on current evidence, mothers and obstetricians need to weigh the benefits of reducing a minimal number of catastrophic outcomes against the harms of increasing minor long‐term adverse outcomes in a substantial number of the general population of children born (Figure 1).

## 2 Advances in the Use of Observational Studies

As reviewed, a range of observational studies explore the association between delivery timing and long‐term cognitive, ADHD, or academic outcomes. However, essential improvements still need to be made in the analyses of observational studies. No meta‐analysis analysed the combined effect of gestational age and birth weight. The most recent meta‐analysis [2] of observational studies indicated that the studies either investigated the impact of gestation or birth weight on the developmental outcome, and a few controlled for one or the other in the analysis. When analysed separately, compared to appropriate for gestational age children (AGA), children born small for gestational age (SGA) had lower cognitive scores (−5.10 IQ points) [7].

In contrast, being large for gestational age (LGA) compared to AGA may have a slight protective effect (+0.90 IQ points) [2]. Only one recent observational study utilising four prospective cohort studies considered the conjoint effects of gestation and birthweight across the whole gestation range [8]. They found that being of lower gestational age and decreasing birthweight for gestational age had additive adverse effects on cognitive outcome. Thus, firstly, future observational studies need to consider the conjoint effect of gestation and birth weight for gestation in term‐born and whether the associations are linear or curvilinear. Secondly, with the greater acceptance of the public for collection of genetic material and reduced costs of genotyping, newer observational studies are likely to determine whether gestation or birth weight are causally associated with neurodevelopmental outcomes or whether polygenetic effects on gestation or birth weight partly or wholly explain these. Thus, genes that are associated with lower gestation may also be associated with neurodevelopmental outcomes [9]. This may even allow the use of Mendelian Randomisation designs to test for causal effects of gestation.

## 3 The Need for Long‐Term Follow‐Up in Obstetrical Clinical Trials

One reason for focusing on short‐term outcomes in RCTs is to provide quick evidence to inform clinical decision‐making. Another reason is the reluctance of funders to provide the required financial support to conduct longer‐term outcome assessments for babies and mothers. However, only large RCTs that can manipulate the delivery timing and have longer‐term follow‐up until school age can determine the trade‐offs of rare catastrophic events or short‐term perinatal benefits compared to the minor adverse long‐term effects of early‐term delivery for many children. Combined with health economic evaluations, it should be able to quantify the trade‐offs.

With increasing sets of more accurate or reliable data ranging from predicted foetal size and pregnancy complications to social and demographic variables, AI and machine learning–aided prediction models may help obstetricians and parents develop personalised birth plans for the timing of birth rather than having to follow one‐size‐fits‐all treatment guidelines.

## Author Contributions

D.W.: conceptualisation, manuscript writing, editing, funding acquisition. X.Z.: conceptualisation, manuscript writing, editing, funding acquisition. S.Q.: conceptualisation, additional input, funding acquisition.

## Conflicts of Interest

The authors declare no conflicts of interest.

## Data Availability

The authors have nothing to report.
